# Radiological assessment of different monazite grades after mechanical separation from black sand

**DOI:** 10.1038/s41598-023-42287-8

**Published:** 2023-09-16

**Authors:** N. A. kotb, M. S. Abd El Ghany, Ashraf A. El-sayed

**Affiliations:** 1 Radiation Protection Department, Hot Labs Center, Egyptian Atomic Energy Authority, Cairo, Egypt; 2 Nuclear Materials Authority, Cairo, Egypt; 3 Analytical Chemistry Department, Hot Labs Center, Egyptian Atomic Energy Authority, Cairo, Egypt

**Keywords:** Environmental sciences, Natural hazards, Chemistry, Physics

## Abstract

In Egyptian black sands, monazite is a precious mineral characterized by its composition, which includes crucial constituents such as thorium, trace amounts of uranium, and rare earth elements. It is essential to evaluate and quantify the extent of gamma-ray exposure resulting from the presence of primordial radionuclides. This necessity arises from human activities that extract and retrieve raw materials in uranium and thorium mining operations. The current study focuses on the radiological assessment of Monazite raw material in various grades and calculates the associated hazard indices. A hyper pure Germanium detector (HPGe) determined the particular activity. For grade, 90% Monazite samples, the average activities for ^232^Th, ^238^U, and ^40^K were 348,008 ± 1406, 69,299 ± 2086, and 27,510 ± 245 Bq/kg, respectively. For grade 75% Monazite samples, the average activities were 219,000 ± 901, 55,000 ± 500, and 18,300 ± 86 Bq/kg, while for grade 50% Monazite samples, it was 43,294 ± 1549, 9593 ± 629, and 4000 ± 211 Bq/kg for the same element, respectively. Also, ^138^La’s inherent radioactivity was taken into account. The computed effective and absorbed dosages exceed the worker’s exempt limit of 20 mSv/y. The calculated hazard parameters are higher than the maximum recommended limits. Therefore, it is imperative to employ radiation safety measures to mitigate the potential hazards of ionizing radiation.

## Introduction

All organisms are consistently subjected to ionizing radiation from naturally occurring radioactive materials (NORM), technically enhanced naturally occurring radioactive materials (TENORM), artificial radionuclides, or nuclear incidents^1^. Several research studies have investigated radiation levels and the distribution of radionuclides in the environment. These studies play a crucial role in providing essential radiological information^1–20^. This knowledge holds excellent importance in comprehending human exposure to radiation occurring from both natural and artificial sources. Additionally, developing guidelines and legislation for radiation protection programs is crucial.

Human behaviors can alter how individuals are exposed to natural radiation sources. Specifically, the release of natural radionuclides into the environment occurs through mineral processing and other applications, such as the manufacturing and utilization of phosphate fertilizers and the combustion of fossil fuels. These activities contribute to increased levels of natural radiation exposure. Many individuals are also subjected to elevated quantities of naturally occurring radiation in their occupational environments. These workers encompass individuals engaged in underground mining, people involved in the processing of minerals, and members of aircraft flight crews^21^.

Beach sands are mineral deposits that are generated through a combination of weathering and erosion processes. These deposits mainly consist of minerals such as quartz and feldspar. The potential origin of these materials in the locations might be attributed to their transportation via wind, rivers, and glaciers, followed by their subsequent deposition on the beaches through the combined effects of waves and currents. The level of natural radioactivity in black stony sand is primarily influenced by the presence of radiogenic heavy mineral deposits in the underlying bedrock. The formation of these deposits is, in turn, affected by the specific geological and geographical conditions of the local area^12^. The utilization of sand or soil has been identified as a significant source of radiation dangers, constituting a prominent contributor to the external dosage of natural radiation for the global population.

Therefore, the radiological consequences can be comprehensive by acquiring knowledge regarding the distribution patterns of the ^238^U and ^232^Th series and ^40^K. Furthermore, the measurement of radioactivity concentration in beach sand can yield significant insights into the mechanisms of movement and the environmental destiny of radionuclides. This information is crucial for assessing the potential health hazards to nearby regions and is vital for establishing a comprehensive and enduring system for monitoring and evaluating radiation levels^12^.

This study focuses on extracting Monazite, a naturally occurring mineral found in sea sand from the Abu-Khashaba area near Rosetta’s north bank of the Nile. Monazite mainly consists of lanthanum elements (Ce, La, Y, Th) PO_4_ alongside cerium, yttrium, and thorium. The composition of Monazite generally consists of approximately 55.0–60% rare earth metal oxides, 24.0–29.0% phosphate (P_2_O_5_), 0.2–0.4% uranium oxide (U_3_O_8_), and 5.0–10.0% thorium oxide (ThO_2_). The thorium ratio in the monazite resource ranges from 6 to 10%. Furthermore, it should be noted that there exist fluctuations in the uranium ratio ranging from 0.20% to 1.0%, as reported in previous studies^22–24^. Egyptian Monazite is a noteworthy rare earth resource, alongside other heavy minerals such as zircon, rutile, and ilmenite^25^.

The current study investigates the radiological evaluations of primordial radionuclides, including ^40^K, in different grades of Monazite. The study aimed to assess the potential risks of radiation exposure on operational personnel and workers during various processing stages. Consequently, multiple radiation hazard indices were identified and analyzed.

## Materials and methods

### Sample collection

**Figure 1:**
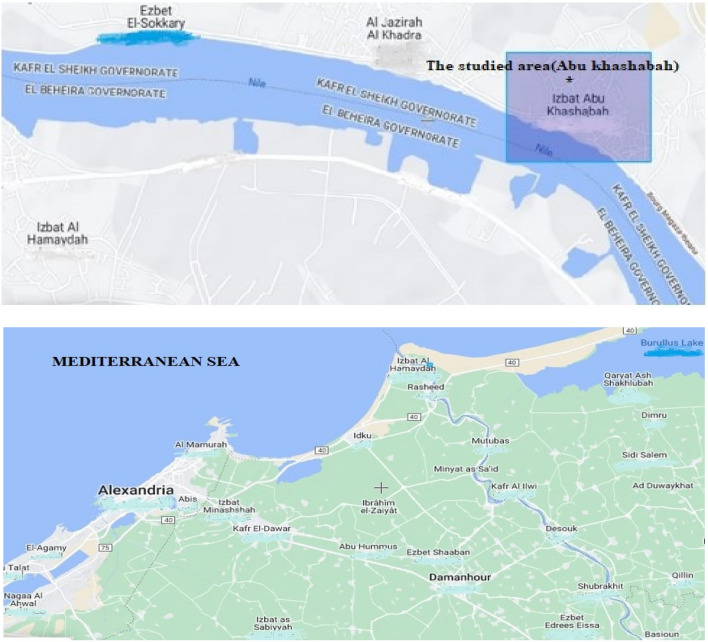
The geographical representation of the area under investigation (Abu Khashabah) region along the Mediterranean coastline.

**Figure 2:**
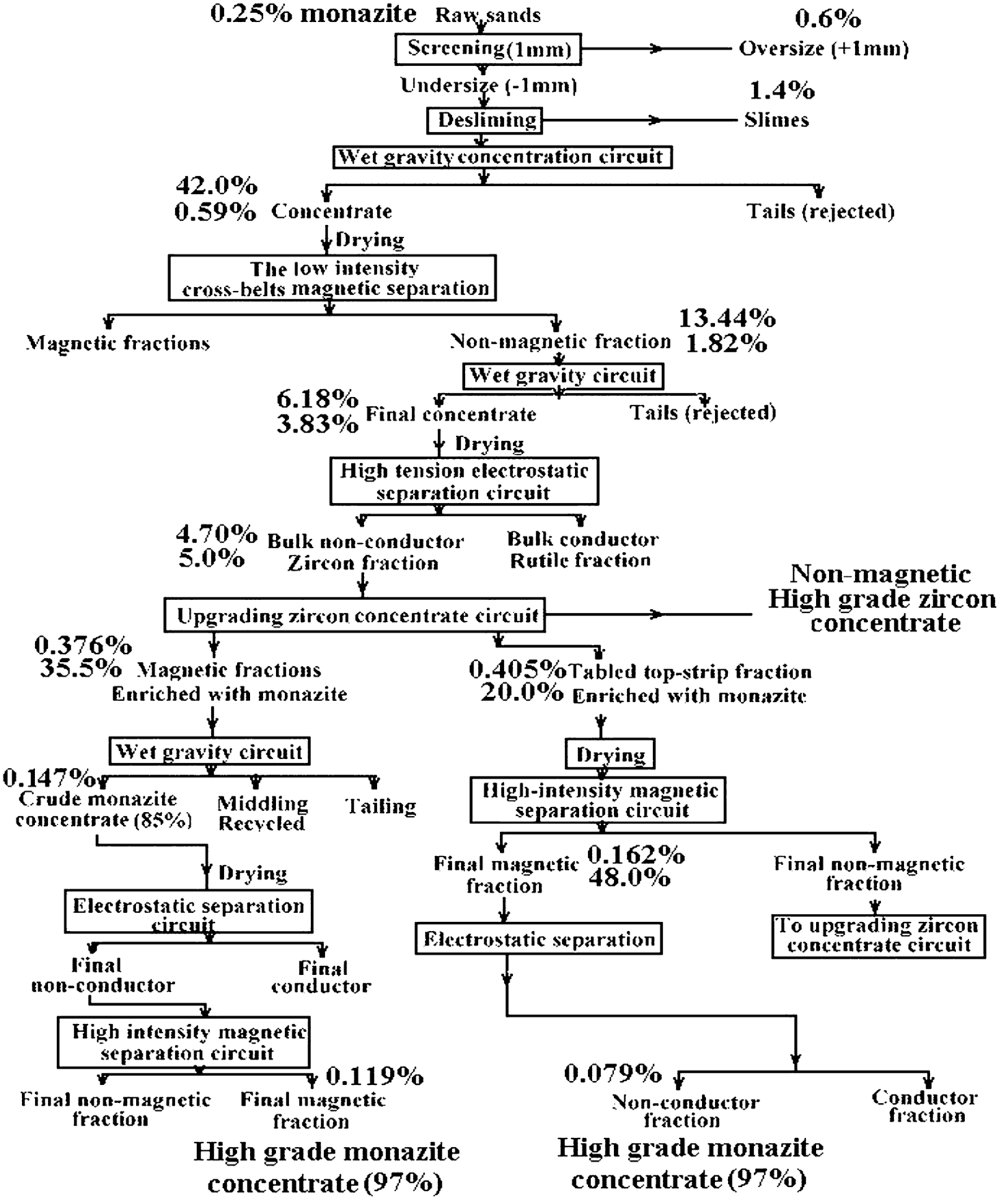
The above flow sheet outlines the procedure for concentrating and separating monazite samples of varying grades.

The raw sand samples were collected from a black sand beach on the Mediterranean coastline near Abu Khashabah, approximately 7 km east of the Rosetta estuary. The provided sample consists of a 2-km segment of unprocessed sand, exhibiting a range of widths extending from a few meters to 20 m. A quantity of sand ranging from 10 to 30 cm was manually removed from the surface. The geographical representation of the area under investigation is depicted in Fig. 1. The image of the map was acquired via the online tool Scribble Maps (https://m.scribblemaps.com/)^26^. The sample was processed by examining physical disparities among the commercially available minerals included in black sand^27,28^. Figure 2 illustrates the Physical Techniques employed for the Separation of Monazite and other Valuable Minerals from Black Sands. Following the concentration and separation of monazite samples with varying grades, the samples were suitably prepared for gamma-ray measurements using an HPGe detector.

### Measurement procedures

The Monazite samples examined in this study were meticulously prepared for laboratory measurements. It underwent a process of air-drying, grinding, and subsequent sieving to achieve a particle size of 200 mesh. Three samples were tested for each grade of Monazite, and the following measurements were conducted. The resulting average value was calculated. The samples were subjected to a sealing process with a duration of 4 weeks to achieve a state of secular equilibrium. This equilibrium is characterized by the daughter radionuclides undergoing disintegration at a rate equivalent to that of the parent radionuclides. Implementing this method is crucial to ensure the adequate confinement of decay products and radon gas within the container and the sample. The containers utilized for the analyzed samples were identical in design to those employed for the reference material during efficiency calibration, ensuring consistent geometric properties.

The gamma-ray activity was determined using a gamma-ray spectrometry system comprising a high-resolution n-type HPGe detector, specifically the GR3021 Canberra model with Serial number 2966076. This detector is designed with a reverse electrode closed-end coaxial geometry, featuring a diameter of 55.5 mm, a length of 52.5 mm, and a distance of 5 mm from the window. The detector has a relative efficiency of 30% and a resolution of 2.1 keV (FWHM) at the energy line of 1.33 MeV. The cryostat model used is 7500SL, the preamplifier model is 2002CSL, and the multi-channel analyzer (MCA) model is MP2-1U with a serial number of 05040700. The data analysis is performed using Genie 2000 software. The placement of the detector occurred within a cylindrical chamber that was insulated with lead, including a permanent bottom and a portable cover at the top. This arrangement was implemented to mitigate the impact of background radiation effectively. The samples were subjected to a counting process lasting 22 h, during which the background counts were also recorded for the same duration to determine the net count. The spectra that were acquired were analyzed utilizing the Genie 2000 software.

The energy calibration of the HPGe detector was conducted by employing certified sealed point sources obtained from Amersham, England. These sources included various emitters, such as ^60^Co (with energies of 1173.2 and 1332.5 keV), ^137^Cs (with an energy of 661.7 keV), and ^226^Ra (with energies ranging from 186 to 2450 keV). These sources were selected based on their wide energy range, making them suitable for calibration. The certified reference materials required for calibrating the efficiency of measuring ^232^Th, ^238^U, and ^40^K activity were obtained from the International Atomic Energy Agency. The materials under consideration include RGTh-1, RGU-1, and RGK-1.

The average activities of ^212^Pb (238.6 keV), ^208^Ti (583.1 and 2614.7 keV), and ^228^Ac (911.1 and 968.9 keV) in the samples were utilized to determine the activity of ^232^Th. The concentration of ^238^U was estimated using the average activity of the decay products ^214^Pb (295.2 and 351.9 keV) and ^214^Bi (609.3, 1120, and 1764.5 keV). The concentration of the radionuclide ^40^K was determined using the gamma line of 1460 keV. The equation utilized to calculate the radioactivity present in the Monazite samples was as follows^5,19^:

1 $${\text{A}}\left( {\text{Bq/kg}} \right){ = }\frac{{{\text{C}}_{{\text{n}}} }}{{{\upvarepsilon }_{{{\gamma }}} {\text{P}}_{{\upgamma }} {\text{ t m}}}}$$

In the given context, the variables are defined as follows: A denotes the activity of a particular nuclide measured in Bq/kg, C_n_ represents the net area counts observed in the corresponding photo peak after background subtraction, ɛ_γ_ signifies the absolute efficiency at the energy of the photo peak, P_γ_ denotes the probability of gamma-ray emission that matches the intensity of the photo peak energy, t represents the duration of counting in seconds (79,200 s), and m represents the mass of the samples in kilograms.

## Results and discussion

### Activity calculation

The radioactivity concentrations of various grades of monazite ore were determined by measuring the gamma rays released by the primordial radionuclides in the ore. Three samples were chosen from each monazite grade and subjected to gamma spectroscopic analysis to evaluate their radioactivity. The resulting measurements were then averaged for each set of three samples Table 1 displays the recorded activity of the primordial radionuclides ^232^Th, ^238^U, and ^40^K in the examined materials. The activity for each grade is determined by calculating the average of three samples. The levels of radioactivity exhibited by the isotopes ^232^Th, ^238^U, and ^40^K are found to be significantly elevated in all samples of Monazite. These samples demonstrate varying degrees of concentration, ranging from approximately 50% for lower grades to 90% for higher grades, with an intermediate grade of 75% observed in certain Monazite specimens. The average activity of ^232^Th, ^238^U, and ^40^K in grade 90% Monazite samples was 348,008 ± 1406, 69,299 ± 2086, and 27,510 ± 245 Bq/kg, respectively. The average activity of Monazite samples with a grade of 75% was found to be 219,000 ± 901, 55,000 ± 500, and 18,300 ± 86 Bq/kg. In contrast, Monazite samples with a grade of 50% exhibited an average activity of 43,294 ± 1549, 9593 ± 629, and 4000 ± 211 Bq/kg. The recorded activity values in all samples exceed the established exemption levels of 35, 30, and 420 Bq/kg for ^232^Th, ^238^U, and ^40^K, respectively^21^. The pattern that has been observed indicates a positive correlation between the grade of the Monazite samples and the concentration of radioactivity. Furthermore, it is seen that the levels of ^232^Th activity are consistently higher than those of both ^238^U and ^40^K in all analyzed samples. The high concentration of thorium (Th) relative to uranium (U) in Monazite is attributed to its composition. Specifically, Monazite typically has 0.2–0.4% uranium oxide (U_3_O_8_) and 5.0–10.0% thorium oxide (ThO_2_) in its component ratios. The Monazite resource has a range of thorium ratios from 6 to 10%. While variations in the uranium ratio range from 0.20 to 1.0% exist^22–24^.

**Table 1:** The specific activity of ^232^Th, ^238^U, and ^40^K in monazite samples is measured in becquerels per kilogram (Bq/kg).

Sample ID monazite	Specific activity (Bq/kg)		
	^232^Th	^238^U	^40^K
Grade 50%	43,294 ± 1549	9593 ± 629	4000 ± 211
Grade 75%	219,000 ± 901	55,000 ± 500	18,300 ± 86
Grade 90%	348,008 ± 1406	69,299 ± 2086	27,510 ± 245

The data for ^232^Th, ^238^U, and ^40^K concentrations in parts per million (ppm) and percentages (%) were derived using the conversion factor documented in the current literature. In the case of ^232^Th, the conversion factor is 1 part per million (ppm) equals 4.06 Becquerels per kilogram (Bq/kg). Similarly, for ^226^Ra, the conversion factor is one ppm equals 12.35 Bq/kg. Lastly, for ^40^K, the conversion factor is 1 percent equals 313 Bq/kg^18,19,29^. For a grade of 90%, the concentration values (in parts per million) are 85,716 ppm for ^232^Th, 5611 ppm for ^238^U, and 88% for ^40^K. The obtained grade is 75%. The measured values for the isotopes ^232^Th, ^238^U, and ^40^K are 54,000, 4454 ppm, and 59%, respectively. The obtained grade is 50%, with the measured values of 10,664 and 777 parts per million (ppm) for the isotopes ^232^Th and ^238^U, respectively. Additionally, the isotope ^40^K was found to have a concentration of 13%.

Based on the assessment of ^138^La’s inherent radioactivity, its decay behavior can be described as follows:

$${}_{57}^{138} La \to {}_{56}^{138} Ba\;\;\left( {{\text{EC}}, {\text{E}}_{\gamma } = { 1435}.{\text{8 keV}},{ 66}.{4}\% } \right)$$

$${}_{57}^{138} La \to {}_{58}^{138} Ce\;\;\left( {\beta^{ - } {\text{Decay}},{\text{ E}}_{\gamma } = { 788}.{\text{7 keV}},{ 33}.{6}\% } \right)$$

The visibility of the two gamma lines of ^138^La at 788.7 and 1435.8 keV in the spectrum of Monazite, namely the lines at 786.3 and 1434.1 keV of ^234^ Pa, may have been hindered by the interference caused by the gamma lines of both daughters of ^232^Th (782.1, 1434.2, and 1438 keV of ^228^Ac) and ^238^U. However, the La sample obtained from the Monazite exhibited distinct visibility of the two gamma lines^27–29^. The activity of ^138^La is around 400 Bq/kg.

### Radiological effects

Various radiation parameters were estimated based on the concentrations of radioactivity, To gain insight into the radiological risks associated with the Monazite samples under investigation. These included the radium equivalent activity (Ra_eq_), The gamma-absorbed dose rate (DR), The annual effective dose rate (AEDR), the external hazard index (H_ex_), the internal hazard index (H_in_), representative gamma level index (I_γr_), The alpha index (I_α_), and the annual gonadal dose equivalent (AGDE).

The radium equivalent activity (Ra_eq_) is a widely used parameter for assessing the radiological effects of naturally occurring radionuclides such as ^226^Ra, ^232^Th, and ^40^K. It comprehensively measures the radiation hazards associated with these elements by combining their activities into a single quantity. The Ra_eq_ parameter is related to assessing both external gamma exposure and internal dosage resulting from radon and its progeny. The Ra_eq_ model is based on the premise that the levels of gamma radiation dosage resulting from 1 Bq/kg of ^226^Ra, 0.7 Bq/kg of ^232^Th, and 13 Bq/kg of ^40^K are considered equal. The subsequent content presents the index:

2 $${\text{Ra}}_{{{\text{eq}}}} {\text{ = C}}_{{{\text{Ra}}}} { + 1}{\text{.43C}}_{{{\text{Th}}}} { + 0}{\text{.077C}}_{{\text{k}}} {,}$$

The abbreviations C_Ra_, C_Th_, and C_K_ indicate the average activity in Bq/kg of ^226^Ra, ^232^Th, and ^40^K, respectively. The obtained results for the radium equivalent (Ra_eq_) of monazite grades at 50%, 75%, and 90% are 71,798, 369,556, and 568,719 Bq/kg, respectively. The estimated values exceeded the allowable limit of 370 Bq/kg as suggested by the International Atomic Energy Agency (IAEA)^16,21,30^. Two supplementary parameters were constructed to represent the gamma-absorbed and annual effective dose rates, and their corresponding values were presented in Table 2. The gamma-absorbed dose in the open air or at a height of one meter above the ground was determined for the examined materials by utilizing the specific activity values of ^40^K, ^226^Ra, and ^232^Th. The computation was executed using the following formula^21^;

3 $${\text{D }}\left( {{\text{nGyh}}^{{ - {1}}} } \right){ = 0}{\text{.0414C}}_{{\text{K}}} { + 0}{\text{.461C}}_{{{\text{Ra}}}} { + 0}{\text{.623 C}}_{{{\text{Th}}}}$$

**Table 2:** The estimated absorbed dose rates nGy/h and the annual effective doses μSv/yr for Monazite samples.

Sample ID monazite	Absorbed dose rates	Annual effective doses
	nGy/h	µSv/yr
Grade 50%	31,564.6	38,710.8
Grade 75%	162,537.2	199,335.6
Grade 90%	249,730.8	306,269.9
Permissible	57	20,000

The investigated samples’ gamma absorbed dose rates for Monazite with grades 50%, 75%, and 90% are 31,564.6, 162,537.2, and 249,730.8 nGy/h. These values exceed the acceptable limit of 57 nGy/h, as established by the United Nations Scientific Committee on the Effects of Atomic Radiation^21^. Depending on the values of gamma absorbed dose rates, The annual effective dose rate was calculated in units of µSv/yr using the formula below^21^;

4 $${\text{Annual Effective }}\;{\text{dose }}\;{\text{rate }}\left( {\mu {\text{Sv}}/{\text{yr}}} \right) \, = {\text{Dose }}\;{\text{rate }}\left( {{\text{nGy}}/{\text{h}}} \right) \, \times { 876}0{\text{ h }} \times \, 0.{2 } \times \, 0.{\text{7 Sv Gy}}^{{ - {1}}} \times \, \left. {{1}0^{{ - {3}}} } \right)$$

The determined annual effective radiation rate exceeds the established worker exemption level defined by the International Commission on Radiological Protection (ICRP)^31^. The significantly increased value can be attributed to the high activity of Monazite.

Two additional parameters signify the risks associated with external and internal radiation. The external hazard index (H_ex_) value must remain below unity to provide a minimal level of radiation hazard. Its formula is^31^;

5 $${\text{H}}_{{{\text{ex}}}} { = }\frac{{{\text{A}}_{{{\text{Ra}}}} }}{{{370}}}{ + }\frac{{{\text{A}}_{{{\text{Th}}}} }}{{{259}}}{ + }\frac{{{\text{A}}_{{\text{k}}} }}{{{4810}}} \le {1}$$

On the other hand, the internal hazard index (H_in_) quantitatively measures internal exposure to carcinogenic radon and its short-lived progeny. The calculation is performed using the subsequent formula:

6 $${\text{H}}_{{\text{in = }}} \frac{{A_{U} }}{{{185}}}{ + }\frac{{A_{Th} }}{{{259}}}{ + }\frac{{A_{K} }}{{{4810}}} \le {1}$$

The variables A_Ra_, A_Th_, and A_K_ denote the activity of ^226^Ra, ^232^Th, and ^40^K, respectively, measured in (Bq/kg). The potential radiation threat should remain minimal if the two index values are less than unity. Based on the data shown in Table 3, it can be observed that the calculated H_ex_ and H_in_ values for the monazite samples exceed a value of one. A supplementary index known as the gamma index (I_γ_) has been formulated to account for the collective effects of ^226^Ra, ^232^Th, and ^40^K in terms of their radiological hazards concerning the exposure of employees and the general public to naturally occurring radioactive materials (NORM). The values for the I_γ_ index were estimated by employing the formula described in references^32,33^.

7 $${\text{I}}_{{\upgamma }} = \frac{{A_{U} }}{300Bq/Kg} + \frac{{A_{Th} }}{200Bq/Kg} + \frac{{A_{K} }}{3000Bq/Kg}$$

**Table 3:** Different hazard indexes Ra_eq_, H_ex_, H_in_, I_γ_, I_α_, and AGED in Monazite samples.

Sample ID monazite	Ra_eq_	H_ex_	H_in_	I_γ_	I_α_	AGED
	Bq/kg					mSv/y
Grade 50%	71,798	193.9	220.9	249.7	102	211.2
Grade 75%	369,556	977.9	1146.6	1284.3	550	1087.8
Grade 90%	568,719	1535.7	1722.2	1979	835	1671.5
Permissible	370	≤ 1	≤ 1	≤ 1	≤ 1	0.3

If the gamma index values exceed unity, the effective dosage received by workers or the general population will be higher than one mSv/y^33^. Based on the data presented in Table 3, it can be observed that the expected values of the gamma index for all the samples analyzed remain higher than one, indicating statistical significance. Another index, the alpha index, focuses on the potential risks associated with inhaling short-lived decay products of ^222^Rn. This issue is of significant concern with indoor radiation exposure^1^. The alpha index (I_α_) is utilized to assess the internal risk arising from the alpha activity of a substance and is expressed as the following numerical value:

8 $${\text{I}}_{\alpha } = \frac{{A_{Ra} }}{200}$$

where A_Ra_ is the concentration of ^226^Ra’s activity Bq/kg. I_α_ must be less than unity for safety. The average values of I_α_ for the measured samples at grades 90%, 75%, and 50% are 835, 550, and 102 Bq/kg, respectively. To evaluate the influence of the activity on organs such as the gonads, active bone marrow, and bone surface cells, it is necessary to quantify the annual gonadal dose equivalent (AGDE) in millisieverts per year (mSv/y). It was determined using the methodology outlined by^34^.

9 $${\text{AGDE = 3}}{\text{.09A}}_{{{\text{Ra}}}} { + 4}{\text{.18A}}_{{{\text{Th}}}} { + 0}{\text{.314A}}_{{\text{K }}} \le { 0}{\text{.3}}\;{\text{mSv/y}}$$

The AGDE factor was calculated to be 1671.5 mSv/y, 1087.8 mSv/y, and 211.2 mSv/y for grade levels of 90%, 75%, and 50%, respectively. These values are significantly more than the worldwide average value for AGDE 0.3 mSv/y^21^.

Based on the data derived from this study concerning the activity levels of ^232^Th, ^238^U, and ^40^K and their comparison with previous research on Egyptian Monazite and Monazite samples from other nations, as presented in Table 4, it is evident that the recorded activity values exceed the acceptable level for radiation safety exemption. Therefore, it is imperative to examine radiation safety measures to mitigate the potential adverse radiological consequences associated with rare earth elements derived from Egyptian Monazite. The radiation protection must be applied so that the magnitude of individual doses must be as low as reasonably achievable, with economic and social factors being considered. Individual dose assessment for workers required the estimation of levels of exposure by applying personal monitoring and workplace monitoring^35^.

**Table 4:** Shows the activity of ^232^Th, ^238^U, and ^40^K of Egyptian Monazite compared with some countries.

Country	Activity (Bq/kg)			References
	^232^Th	^238^U	^40^K	
Egypt	442,105 ± 29,200	54,435 ± 3138	5841 ± 345	^5^
	142,850 ± 1940	Ra-226 (36,530 ± 290	< MDA	^17^
		Pb-210 (2890 ± 160)		
	43,294 ± 1549	9593 ± 629	4000 ± 211	Present study (grade50
	219,000 ± 901	55,000 ± 500	18,300 ± 86	Present study (grade75)
	348,008 ± 1406	69,299 ± 2086	27,510 ± 245	Present study(grade90)
Australia	Ra-228	Ra-226	–	^36^
	242,000	30,000	–	
Malaysia	62,000.410 ± 0.05	17,000.980 ± 0.24	–	^2^
Malaysia	1150 ± 800	500 ± 300	–	^3^
World average	35	30	420	^21^

## Conclusion

The present study aims to examine the radiological assessments of primordial radionuclides in various grades of Monazite. The activity levels of ^232^Th, ^238^U, and ^40^K in all the analyzed monazite samples were higher than those considered exempt. All computed radiological hazard parameters exceed the allowable limits. Hence, it is imperative to mandate radiation safety protocols throughout the processing of Monazite for the extraction of industrially and nuclearly relevant metals, such as rare earth elements (REE_s_), thorium (Th), and uranium (U). To ensure the safety of workers involved in processing raw materials, it is imperative to implement a radiation safety program that adheres to the following guidelines: the justification of practices, the limitation of radiation doses to individuals, and the optimization of radiation protection. The acquired data can be employed as a repository to oversee the natural radioactivity of monazite processing and its corresponding index of radiological dangers.

## Data Availability

The datasets used and/or analysed during the current study available from the corresponding author on reasonable request.

## References

[1] El-Afifi EM, Shahr-El-Din AM, Aglan RF, Borai EH, Abo-Aly MM (2017). Baseline evaluation for natural radioactivity level and radiological hazardous parameters associated with the processing of high-grade Monazite. Regul. Toxicol. Pharmacol..

[2] Al-Areqi WM, Bahri CN, Majid AA, Sarmani S (2016). Separation and radiological impact assessment of thorium in Malaysian monazite processing. Malays. J. Anal. Sci..

[3] Omar M, Hassan A (2002). The occurrence of high concentrations of natural radionuclides in the black sands of Malaysian beaches. JSNM.

[4] Iwaoka K, Yajima K, Suzuki T, Yonehara H, Hosoda M, Tokonami S, Kanda R (2017). Investigation of natural radioactivity in a monazite processing plant in Japan. Health Phys..

[5] Hamed MM, Hilal MA, Borai EH (2016). Chemical distribution of hazardous natural radionuclides during monazite mineral processing. J. Environ. Radioact..

[6] Shuaibu HK, Khandaker MU, Alrefae T, Bradley DA (2017). Assessment of natural radioactivity and gamma-ray dose in Monazite rich black sand beach of Penang Island, Malaysia. Mar. Pollut. Bull..

[7] Admola JA, Nwafor CO (2013). Radiological Risk Assessment of Natural Radionuclides in Sand Collected from some beaches along the coastline of southwestern Nigeria. Radiat. Prot. Dosim..

[8] Freitas AC, Alencar AS (2004). Gamma dose rates and distribution of natural radionuclides in sand beaches-Ilha Grande, Southeastern Brazil. J. Environ. Radioact..

[9] Hassan AM, Abdel-Wahab M, Nada A, Walley-El-Dine N, Khazbak A (1997). Determination of uranium and thorium in Egyptian Monazite by gamma-ray spectrometry. Appl. Radiat. Isotopes.

[10] Uosif MAM, El-Taher A, Abbady GEA (2008). Radiological significance of beach sand used for climate therapy from Safaga, Egypt. Radiat. Prot. Dosim..

[11] Khandaker MU, Asaduzzaman K, Bin Sulaiman AF, Bradley DA, Isinkaye MO (2018). Elevated concentrations of naturally occurring radionuclides in heavy mineral-rich beach sands of Langkawi Island, Malaysia. Mar. Pollut. Bull..

[12] Khandaker MU, Garba NN, Rohaizad CAHBC, Bradley DA (2019). Assessment of natural radioactivity levels in stony sand from Black Stone Beach of Kuantan, the Peninsular Malaysia. Radioprotection.

[13] Ali MMM, Zhao H, Rawashded A, Mohammed YA, Al-Hassan M (2021). Assessment of radiation hazard indices for sand samples from Marib in Yemen. Int. J. Radiat. Res..

[14] Emad BM, Sayyed MI, Somaily HH, Han MY (2022). Natural radioactivity and radiological hazard effects from granite rocks in the Gabal Qash Amir Area, South Eastern Desert, Egypt. Minerals.

[15] Orlando M, Passamai JL, Zordan AB (2022). Physicochemical characterization of monazite sand and its associated bacterial species from the beaches of southeastern Brazil. Environ. Sci. Pollut. Res..

[16] Mireles F, Davila JI, Quirino LL, Lugo JF, Pinedo JL, Rios C (2003). Natural soil gamma radioactivity levels and resultant population dose in the city of Zacatecas and Guadalupe, Zacatecas, Mexico. Health. Phys..

[17] Borai EH, El-Afifi EM, Shahr-El-Din AM (2017). Selective elimination of natural radionuclides during the processing of high grade monazite concentrates by caustic conversion method. Korean J. Chem. Eng..

[18] Adewoyin OO, Maxwell O, Akinwumi AA, Adagunod TA, Embong Z, Saeed MA (2022). Estimation of activity concentrations of radionuclides and their hazard indices in coastal plain sand region of Ogun state. Sci. Rep..

[19] Belyaeva O, Pyuskyulyan K, Movsisyan N, Saghatelyan A, Carvalho FP (2019). Natural radioactivity in urban soils of mining centers in Armenia: Dose rate and risk assessment. Chemosphere.

[20] Abd-Elkader MM, Shinonaga T, Sherif MM (2021). Radiological hazard assessments of radionuclides in building materials, soils and sands from the Gaza Strip and the north of Sinai Peninsula. Sci. Rep..

[21] United Nations Scientific Committee on the Effects of Atomic Radiation (UNSCEAR). Sources and effects of ionizing radiation. In *Report to the General Assembly with Annexes. United Nations, New York* (2000).

[22] Ja YG (1979). Decomposition of Monazite sand. J. Korean Chem. Soc..

[23] Gupta CK, Mukherjee TK (1990). Hydrometallurgy in Extraction Processes.

[24] Moeller T (1973). The Chemistry of the Lanthanides: Pergamum Texts in Inorganic Chemistry.

[25] Gupta CK, Krishnamurthy N (2005). Extraction Metallurgy of Rare Earth.

[26] Hennig S, Vogler R, Jekel T (2011). Web-2.0 Anwendungen zur partizipativen Planung und Sozialen Geokommunikation. GIS. Sci. Die Zeitschr. Geoinform..

[27] Moustafa MI, Abdelfattah NA (2010). Physical and chemical beneficiation of the Egyptian beach monazite. Resour. Geol..

[28] Helaly, O. S. Separation of Cerium from Egyptian Monazite Sands using Solvent Impregnated Resin. In *Ph. D. Thesis, Chemical Eng. Dept., Faculty of Eng., Cairo University, Egypt* (2011).

[29] Omeje M (2020). Spatial distribution of gamma radiation dose rates from natural radionuclides and its radiological hazards in sediments along river Iju, Ogun state Nigeria. MethodsX..

[30] United Nations Scientific Committee on the Effects of Atomic Radiation (UNSCEAR). In *Exposure from natural sources of radiation. Report to the General Assembly with Annexes*. United Nations. New York (1993).

[31] International Commission on Radiological Protection. Recommendations of the International Commission on Radiological Protection, ICRP Publication 60, Ann. ICRP 21. 1–3, Pergamon Press, Oxford and New York (1991). 2053748

[32] EC, European Commission. Radiological protection principles concerning the natural radioactivity of building materials. In *Radiat. Prot*. **112**, Directorate General Environment, Nuclear Safety and Civil Protection, European Commission, Luxembourg, Belgium (1999).

[33] European Commission (EC). Report on radiological protection principles concerning the natural radioactivity of building materials. In *Radiat. Prot.* **112**, Directorate-General Environment, Nuclear Safety, and Civil Protection, STUK Finland (2000).

[34] United Nations Scientific Committee on the Effects of Atomic Radiation (UNSCEAR). Sources, effects, and risks of ionizing radiation. In *Report to the General Assembly with annexes*. United Nations Publication, New York (1988).

[35] International Atomic Energy Agency (IAEA). Occupational radiation protection in the mining and processing of raw materials. In *Safety Standard Series. No. RS-G-1.6* (2004).

[36] Anvia M, Brown SA, Mc-Orist GD (2015). The deportment of uranium decay chain radionuclides during processing of an Australian monazite concentrate using a caustic conversion route. J. Radioanal. Nucl. Chem..

